# Deep Learning and IoT-Based Ankle–Foot Orthosis for Enhanced Gait Optimization

**DOI:** 10.3390/healthcare12222273

**Published:** 2024-11-14

**Authors:** Ferdous Rahman Shefa, Fahim Hossain Sifat, Jia Uddin, Zahoor Ahmad, Jong-Myon Kim, Muhammad Golam Kibria

**Affiliations:** 1 Department of Computer Science and Engineering, University of Liberal Arts Bangladesh, Dhaka 1207, Bangladesh; ferdous.rahman.cse@ulab.edu.bd (F.R.S.); fahim.hossain.cse@ulab.edu.bd (F.H.S.); golam.kibria@ulab.edu.bd (M.G.K.); 2 Department of AI and Big Data, Endicott College, Woosong University, Daejeon 300718, Republic of Korea; jia.uddin@wsu.ac.kr; 3 Department of Electrical, Electronics, and Computer Engineering, University of Ulsan, Ulsan 44610, Republic of Korea; zahooruou@mail.ulsan.ac.kr

**Keywords:** good health and well-being, ankle–foot orthosis, Internet of Things, wearable device, healthcare, machine learning

## Abstract

Background/Objectives: This paper proposes a method for managing gait imbalances by integrating the Internet of Things (IoT) and machine learning technologies. Ankle–foot orthosis (AFO) devices are crucial medical braces that align the lower leg, ankle, and foot, offering essential support for individuals with gait imbalances by assisting weak or paralyzed muscles. This research aims to revolutionize medical orthotics through IoT and machine learning, providing a sophisticated solution for managing gait issues and enhancing patient care with personalized, data-driven insights. Methods: The smart ankle–foot orthosis (AFO) is equipped with a surface electromyography (sEMG) sensor to measure muscle activity and an Inertial Measurement Unit (IMU) sensor to monitor gait movements. Data from these sensors are transmitted to the cloud via fog computing for analysis, aiming to identify distinct walking phases, whether normal or aberrant. This involves preprocessing the data and analyzing it using various machine learning methods, such as Random Forest, Decision Tree, Support Vector Machine (SVM), Artificial Neural Network (ANN), Long Short-Term Memory (LSTM), and Transformer models. Results: The Transformer model demonstrates exceptional performance in classifying walking phases based on sensor data, achieving an accuracy of 98.97%. With this preprocessed data, the model can accurately predict and measure improvements in patients’ walking patterns, highlighting its effectiveness in distinguishing between normal and aberrant phases during gait analysis. Conclusions: These predictive capabilities enable tailored recommendations regarding the duration and intensity of ankle–foot orthosis (AFO) usage based on individual recovery needs. The analysis results are sent to the physician’s device for validation and regular monitoring. Upon approval, the comprehensive report is made accessible to the patient, ensuring continuous progress tracking and timely adjustments to the treatment plan.

## 1. Introduction

Gait issues, encompassing difficulties walking, are increasingly recognized for their broad impact across age groups and can stem from various causes such as trauma, neurological disorders, musculoskeletal conditions, or aging. These issues significantly curtail mobility, induce discomfort, and diminish overall quality of life, affecting both physical and mental well-being. Moreover, reduced mobility heightens the risk of falls and injuries, exacerbating the challenges faced by individuals experiencing gait problems requiring intricate balance and coordination of lower extremity muscles through a series of repetitive steps and strides termed gait cycles [1]. Comprising two key phases—Stance and Swing—the gait cycle delineates the movement from foot contact to foot liftoff and vice versa, with Stance occupying approximately 60–62% and Swing around 38–40% of the cycle [2]. Any disruption to this cyclic motion characterizing normal gait, leading to non-consecutive phase occurrences, denotes an abnormal gait cycle. Gait difficulties significantly impact the quality of life, restricting both sufferers and caregivers in their daily activities. Various factors, including aging, illness, injury, and neurological conditions such as stroke, cerebral palsy, and paralysis, contribute to gait impairments [3]. Consequent limitations in physical activity elevate the risk of falls and injuries, eroding self-confidence and independence, thereby fostering feelings of frustration and isolation. Moreover, walking impairments can hinder basic daily functions, imposing substantial physical and emotional burdens on caregivers, including personal care, medication management, and mobility assistance, often leading to financial stress and caregiver fatigue.

Notably, lower extremity disorders, encompassing the thigh, leg, ankle, and foot, precipitate numerous illnesses compromising mobility and stability. Symptoms, such as walking disparities, abnormal posture, stumbling, and recurrent falls, signal the presence of these disorders, posing substantial risks of harm, disability, and diminished quality of life [4]. Falls stemming from factors like poor balance and mobility restrictions compound these risks, underscoring the critical importance of addressing gait abnormalities promptly.

Drop-foot, a prevalent condition characterized by the inability to dorsiflex the foot, presents a notable example of abnormal gait. Afflicted individuals compensate by elevating the knee higher during ambulation, resulting in an irregular gait pattern fraught with discomfort, clumsiness, and impairment [5,6]. Prolonged standing or walking exacerbates symptoms, eliciting fatigue and discomfort, with severe cases culminating in the inability to lift the foot, impeding mobility and daily activities. The multifaceted impact of drop-foot on quality of life underscores the imperative of early diagnosis and intervention to mitigate symptom severity and optimize outcomes [7].

Timely medical intervention plays a pivotal role in managing lower extremity disorders, offering a spectrum of treatments tailored to individual needs. Physical therapy, comprising exercises to enhance strength, flexibility, and balance, alongside posture and gait optimization techniques, constitutes a cornerstone of management strategies [8]. Surgical interventions may be warranted in severe cases to address underlying pathologies, while comprehensive rehabilitation programs facilitate functional recovery and enhance the quality of life for individuals with conditions like cerebral palsy and stroke.

To enhance mobility and quality of life, healthcare professionals often recommend the use of assistive devices, including adaptive equipment and orthotics. Among these, ankle–foot orthosis (AFO) stands out as a pivotal intervention for individuals with conditions affecting their lower extremities, offering significant benefits when combined with physiotherapy. Physiotherapy addresses muscle stiffness, while the AFO improves ankle joint movement, providing stability, gait symmetry, and balance during walking [9]. This synergistic approach effectively treats various lower extremity conditions, enhancing overall health, reducing fall risks, and ameliorating complications [10].

The AFO, a custom-fitted orthotic device worn around the ankle and foot, supports these vital joints during movement, thereby preventing and treating a spectrum of lower limb deformities, including pes planus (flatfoot). Flatfoot, characterized by the collapse of the foot arch, leads to pain, instability, and increased fall risk, underscoring the importance of AFO in mitigating such conditions. Moreover, AFO serves as a versatile medical brace capable of addressing foot drop, overpronation, and associated lower extremity pain [11].

Despite the efficacy of ankle–foot orthosis (AFOs) and physiotherapy, gauging patient progress remains challenging, necessitating continuous feedback from physiotherapists. These professionals assess improvements through observations of mobility, stability, and posture, discerning signs of enhancement in patient comfort and function [12]. However, determining the optimal duration of ankle–foot orthosis (AFO) wear poses a dilemma, as recovery timelines vary depending on condition severity and underlying etiology, leaving individuals with gait disorders to endure prolonged discomfort.

The advent of Internet of Things (IoT) technologies heralds a paradigm shift in healthcare delivery, facilitating real-time remote monitoring through wearable devices, service modularity for reusability [13], and user-centric knowledge creation [14] in an IoT environment, and enabling data-driven insights through machine learning algorithms. By leveraging wireless networks and cloud servers, the IoT-enabled AFO empowers physiotherapists to monitor patient progress dynamically, facilitating informed treatment decisions and personalized care adjustments.

Ankle–foot orthoses (AFOs) serve as pivotal walking aids and play a crucial role in ameliorating gait abnormalities and mitigating fall risks. These devices, worn around the foot and ankle, enhance balance, coordination, and muscle flexibility, thereby improving gait speed, endurance, and overall body function [15,16]. While the effectiveness of AFOs in long-term use varies depending on individual circumstances and the severity of underlying conditions, they offer substantial benefits, particularly in correcting foot and ankle deformities like foot drop. Regular evaluation by physiotherapists or physicians is paramount to gauge the extent of improvement achieved with AFO use. However, this process entails significant time and financial commitments, often overwhelming patients and their caregivers. The cumulative costs associated with healthcare appointments, AFO procurement, and related expenses pose considerable challenges, compounded by the logistical burdens of travel and waiting times [17,18,19].

The suggested method incorporates an Internet of Things (IoT)-enabled ankle–foot orthosis (AFO) device that makes use of surface Electromyography (sEMG) and Inertial Measurement Unit (IMU) signals. While the sEMG sensor records muscle electrical activity during movement, the IMU sensor tracks walking patterns. The main objective is to provide a thorough evaluation of the user’s muscle function and mobility. A comprehensive understanding of the user’s gait is ensured by this dual-signal technique, which is essential for precise analysis. The system can recognize patterns and forecast gait improvements with high precision by analyzing these signals using complex machine-learning methods. This integration significantly enhances patient care by enabling real-time, tailored feedback and adaptive support. Representing a significant leap over the most advanced techniques currently available, it provides a more accurate and all-encompassing means of treating gait abnormalities and enhancing patient mobility.

The rest of the paper is organized as follows: state-of-the-art papers are reported in Section 2; the proposed methodology is presented in Section 3; experimental results analysis is provided in Section 4; limitations are discussed in Section 5; and finally, the paper is concluded in Section 6.

## 2. Literature Review

Ankle–foot orthosis (AFO) has been a focal point of research over recent decades, garnering significant attention from experts and scientists [20,21]. Despite the wealth of studies conducted in this domain [22], the integration of gait analysis utilizing AFO within the Internet of Things (IoT) framework remains relatively unexplored. This study aims to bridge this gap by developing a state-of-the-art IoT-based AFO capable of real-time lower limb activity monitoring while supporting and assisting patients’ muscle function and movements [23]. By leveraging the latest IoT advancements, this study seeks to enhance the efficacy of gait analysis, addressing a critical gap in current research and signaling the need for further exploration and investigation in this promising field.

The utilization of ankle–foot orthoses (AFOs) as a therapeutic intervention has gained widespread acceptance for various lower limb conditions, including cerebral palsy [24], stroke [25], and weakened lower limb musculature. Stroke and cerebral palsy, debilitating conditions affecting mobility and muscle tone, underscore the importance of effective treatment modalities to improve walking function and overall quality of life. AFOs provide crucial support, stability, and gait enhancement, facilitating better control over foot and lower limb movements. Their utility extends to correcting deformities associated with clubfoot, drop foot, and various forms of foot and ankle instability.

Conditions such as stroke, multiple sclerosis, and cerebral palsy often manifest in aberrant gait patterns, posing challenges to mobility and increasing fall risks [26]. Ankle–foot orthoses (AFOs) offer a viable solution by ameliorating abnormal gait patterns, enhancing mobility, and reducing fall incidence, thereby enhancing individuals’ ability to engage in daily activities [27]. The diversity of AFO designs and configurations reflects ongoing efforts to tailor interventions to meet the specific needs of gait-impaired patients, fostering improved comfort, security, and overall satisfaction with gait performance.

Rehabilitation constitutes a pivotal component of medical care for individuals grappling with diverse physical and neurological conditions [28]. In recent years, the integration of wearable technology with the IoT has emerged as a promising avenue for enhancing rehabilitation outcomes. In a study by Veeresh Babu et al. [29], a remote monitoring system was developed for lower limb rehabilitation, showcasing the potential of wearable technology in this domain. Numerous studies have underscored the transformative impact of wearable technology on rehabilitation, emphasizing its capacity to improve patient care and consequently elevate the quality of life [30].

Smart AFO exemplifies the integration of wearable technology into rehabilitation protocols, facilitating more precise and efficient rehabilitation activities. Research efforts, such as the experiment conducted by Zhou et al. [31], focusing on ankle–foot movement recognition-based experiments for smart AFO, demonstrate the continuous evolution of technology in this field. The utilization of advanced technology-based AFOs, including IoT-enabled devices, holds promise for enhancing rehabilitation outcomes, promoting patient participation, and improving accessibility [32].

The advent of wearable technology in rehabilitation not only fosters patient engagement and enhances outcomes but also streamlines patient management by providing real-time progress data to healthcare professionals, particularly for patients with conditions such as stroke and cerebral palsy [33]. This wealth of patient data not only informs treatment strategies but also drives further research and innovation in the field [34].

While the field of rehabilitation continues to evolve, with a plethora of IoT-based devices being developed, limited attention has been paid to IoT-based AFO. Despite the advancements in IoT technology, research in IoT-based ankle–foot orthoses (AFOs) remains scarce compared to other domains of IoT and wearable technology. For instance, studies on IoT-based footwear solutions have proliferated, showcasing innovative applications such as real-time patient monitoring and gait tracking [35,36,37].

However, notable exceptions exist, such as the lightweight exosuit developed by Phong et al. [38], which autonomously recognizes gait phases and situations based on IoT principles. Additionally, Wahid et al. [39] utilized 3D printing technology to develop an IoT-based AFO, demonstrating the potential for integration with emerging manufacturing techniques. Similarly, Sabani et al. [40] developed an Android-based monitoring system for leg orthosis, highlighting the versatility of IoT applications in rehabilitation. Despite these strides, further research is warranted to fully harness the potential of IoT-based AFOs in optimizing rehabilitation outcomes.

Despite their potential, sensors such as heart rate and temperature sensors have not fully realized their efficacy in remote gait analysis, necessitating further refinement and development [40,41].

Gait analysis is inherently complex, involving the collection and analysis of diverse data points encompassing muscle activation patterns, posture, balance, and gait velocity. To unleash the true potential of these sensors in remote gait analysis, they must be seamlessly integrated into a cohesive system capable of accurately collecting, analyzing, and interpreting data in real time. The absence of such a comprehensive solution poses a significant challenge for researchers and clinicians striving to enhance rehabilitation outcomes for individuals with gait-related issues. Electromyography (EMG) and Inertial Measurement Unit (IMU) sensors have emerged as pivotal tools in body activity analysis within wearable technology and rehabilitation contexts [42,43]. EMG sensors detect muscle activities, unveiling muscle activation patterns critical for understanding movement dynamics. Conversely, IMU sensors capture lower limb movement, offering insights into posture, balance, and gait velocity. The synergistic integration of these sensors furnishes healthcare providers with a holistic view of patients’ gait and movements, empowering the formulation of more efficacious rehabilitation strategies [44].

Electromyography (EMG), a technique employed to evaluate muscle electrical activity, is instrumental in studying muscle physiology and movement patterns. Surface Electromyography (sEMG), a non-invasive variant, finds widespread utility in various domains, including sports, rehabilitation, and ergonomics. By measuring muscle electrical activity, sEMG facilitates performance, fatigue, and muscle activity assessments, offering invaluable insights into movement dynamics and functional capacity [45].

The procedure for recording electrical signals generated during muscular contractions involves the application of electrodes to the skin surface over the muscle of interest [46,47]. The EMG sensor, a longstanding tool for testing neuromuscular responses, has seen increased utilization in recent years due to the growth of wearable IoT technologies [20,42,48]. EMG sensors have been employed for diverse purposes, from gait event detection to monitoring muscle fatigue. For instance, in [49], researchers utilized sEMG sensor data in conjunction with a Vicon Motion Capture System to identify abnormal gait patterns, albeit at considerable expense. Conversely, studies like that of Morbidoni et al. [50] demonstrated the effectiveness of low-cost sEMG devices in detecting gait phases and events using machine learning algorithms. Additionally, low-cost sEMG devices offer mobility and ease of use, facilitating research in real-world environments and yielding ecologically sound results [51].

Inertial Measurement Unit (IMU) sensors have been pivotal in inertial-based walking navigation systems, offering insights into the gait, motion, and orientation of the lower limbs [52]. IMU sensors provide accurate data on linear and angular acceleration, enabling a comprehensive assessment of posture, balance, and gait speed. The integration of multiple IMU sensors enables detailed analysis of lower limb functionality during movement, which is crucial for biomechanics, sports performance analysis, and rehabilitation [19,53,54,55]. The MPU-6050 IMU sensor (TDK, Sunnyvale, CA, USA) has been particularly prominent in various studies due to its adaptability and precision in capturing complex gait patterns and assessing lower limb movements in real time [56,57].

To collect sensor data, microcontrollers such as Arduino and NodeMCU are commonly utilized, serving as interfaces between sensors and processing units [54]. These microcontrollers, renowned for their small size, low power consumption, and cost-effectiveness, are well-suited for wearable applications, facilitating deeper insights into lower limb functions and movements in rehabilitation and gait analysis [58]. In IoT applications, wireless communication protocols such as Wi-Fi or Bluetooth are frequently employed for transmitting data to the cloud [19,40,52].

Gait analysis, crucial for rehabilitation, increasingly relies on wearable sensors like Electromyography (EMG) and Inertial Measurement Unit (IMU) sensors. Machine learning (ML) and deep learning (DL) methods are now applied to interpret sensor data, enhancing understanding of lower limb mechanics and optimizing patient rehabilitation. Research papers have employed various models, including Decision Tree, Support Vector Machine (SVM), and Artificial Neural Network (ANN), to classify gait phases, recognize abnormal patterns, and differentiate between walking with and without an ankle–foot orthosis (AFO). Notably, LSTM has shown promise in some studies, while BiLSTM has outperformed other models [31]. L. Meng et al. [44] investigated the effects of integrating surface Electromyography (sEMG) data with acceleration data on locomotion prediction. They implemented both machine learning (ML) models, including Support Vector Machine (SVM), and deep learning (DL) models, such as Artificial Neural Networks (ANNs), for this purpose. The authors of [49] implemented both machine learning (ML) and deep learning (DL) to classify and recognize abnormal gait patterns. In [50], Artificial Neural Networks (ANNs) were applied to achieve optimal results in classifying gait phases. While wearable sensors directly mounted on legs have been primarily used, there’s a growing emphasis on developing sophisticated sensors to provide more comprehensive and precise gait analysis as technology evolves [59]. The authors of [60] compare the accuracy of XGBoost, Random Forest, and Support Vector Machine (SVM) for human gait prediction, with Random Forest reaching 99.6%, XGBoost 98.8%, and SVM 98.2%. With advances in gait recognition technology, Transformer-based models are being explored to enhance accuracy in identifying walking patterns. Wu and Zhao [61] improved gait recognition, achieving 99.26% accuracy on the UNTIO normal gait dataset. For Parkinson’s disease gait data, they achieved 98.72% accuracy in identifying healthy controls and 97.53% accuracy for severe cases. Nguyen et al. [62] introduced a Transformer-based model for detecting Parkinson’s disease from gait analysis, achieving 95.2% classification accuracy and 89% accuracy for individual walking segments.

Table 1 provides a clear comparison of the advantages and disadvantages of each technique.

## 3. Design and Development of IoT-Based Smart AFO

The advancements in Inertial Measurement Unit (IMU) sensors and their integration with microcontrollers and wearable technologies have laid a solid foundation for developing innovative solutions in gait analysis and rehabilitation. Leveraging the precision and adaptability of sensors like the MPU-6050, combined with the power of microcontrollers such as Arduino and NodeMCU, enables real-time monitoring and detailed analysis of lower limb movements. These technological components facilitate the creation of IoT-enabled devices that provide valuable insights into gait patterns, which are essential for effective rehabilitation and sports performance analysis. Furthermore, the application of machine learning techniques to interpret sensor data enhances the understanding of lower limb mechanics, enabling more personalized and effective patient care.

In this study, a variety of machine learning (ML) and deep learning (DL) techniques were utilized to classify the dataset. The machine learning models included Random Forest, Decision Tree, and Support Vector Machine (SVM), which are popular for classification tasks due to their effectiveness in managing complex, high-dimensional data. The deep learning models comprised Long Short-Term Memory (LSTM), Artificial Neural Network (ANN), and a Transformer-based model, which excel at identifying intricate patterns and feature interactions within the data. Key parameters for each model were meticulously adjusted to enhance performance. For the Random Forest model, optimization focused on the number of trees and tree depth, while the SVM model was fine-tuned using kernel selection and regularization parameters. In the deep learning models, factors such as the number of layers, neurons per layer, learning rate, and batch size were optimized. The Transformer model, designed for sequence data, included additional parameters like attention heads and projection layers to tailor it for tabular data. These selected parameters played a crucial role in improving the models’ ability to generalize and perform accurately across various algorithms.

Building on these principles, the following sections detail the design and development of an IoT-based Smart AFO, highlighting the integration of various hardware components and their roles in monitoring and improving gait dynamics. The following sub-sections present the details of the design and implementation of the proposed IoT-based framework.

### 3.1. Hardware Description and Integration

The integration of various components, such as sensors, microcontrollers, and actuators, is crucial for the effective functioning of IoT-enabled smart ankle–foot orthosis (AFO). This section offers a comprehensive overview of the hardware implementation process, detailing the different stages and underlying principles. By providing a detailed description of the hardware setup, it aims to provide valuable insights into the design and development of these innovative orthotic devices.

In creating an IoT-based Smart AFO, a range of IoT technology components are integrated to observe the gait patterns of AFO wearers in real time. The hardware components include sensors, microcontrollers, databases, cloud infrastructures, and end devices. A low-cost Muscle Sensor V3 model sEMG sensor, equipped with two surface electrodes, monitors leg muscle activity in real time. It produces an analog output by measuring, filtering, rectifying, and amplifying the electrical activity of muscles. These data are useful for applications ranging from physical rehabilitation to sports science. Concurrently, the MPU-6050 IMU sensor, which is both small and affordable, combines a three-axis gyroscope, an accelerometer, and a digital motion processor. This research focuses on the accelerometer data, which records gait acceleration along the X, Y, and Z axes and communicates via the I2C protocol, providing essential details about the alignment and motion of the lower limbs.

**Figure 1:**
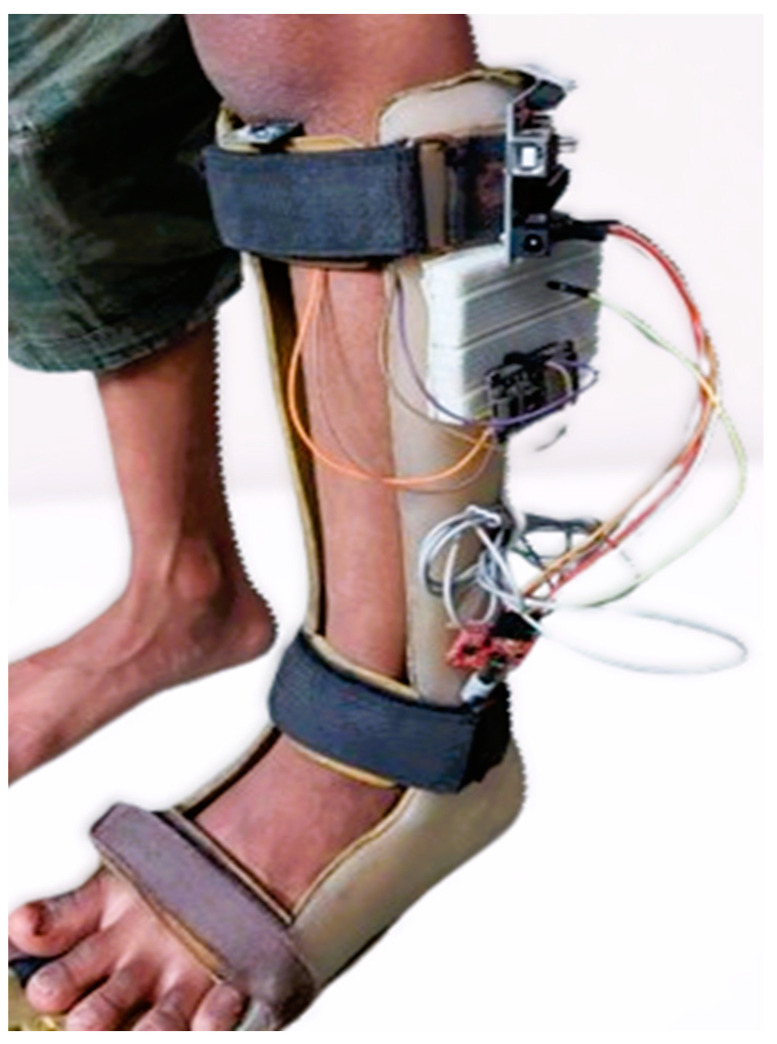
Working Prototype of a Smart AFO on a patient.

Additionally, the Arduino UNO offers a wide range of libraries and components that simplify development. It features 14 digital inputs and outputs (six can be used for PWM outputs), six analog inputs, a 16 MHz ceramic resonator, a USB port, an ICSP header, and a reset button. NodeMCU-ESP-8266 (Espressif Systems, Shanghai, China) is known for its low cost and energy efficiency and supports Wi-Fi networking. The prototype depicted in Figure 1 is effective and easy to use. The IoT device, consisting of an IMU and sEMG sensor, an Arduino UNO, and a NodeMCU, has a combined weight of approximately 95 g. Physiotherapists recommend ankle–foot orthoses (AFOs) to address various gait issues, such as foot drop, spasticity, leg muscle weakness, and paralysis. Constructed from polypropylene sheets (a type of thermoplastic polymer), the AFO is lightweight, durable, chemically resistant, and exhibits excellent impact strength. It is important to note that this AFO is not a one-size-fits-all solution; each patient receives a custom AFO tailored to their height, weight, and muscle condition. When an orthotist creates an AFO, they ensure it meets the specific needs of the patient. For testing the prototype, the device is securely attached to each patient’s custom AFO with tape to guarantee proper fit and functionality. During testing, patients were able to walk without any issues while wearing the prototype. Feedback was frequently solicited regarding any discomfort, and no issues were reported. Had any problems arisen, the prototype would have been immediately removed.

### 3.2. System Architecture

**Figure 2:**
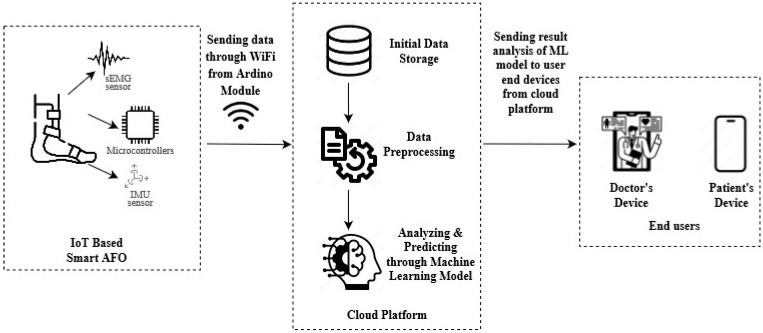
System Architecture of Machine Learning and IoT-Driven Smart AFO.

This system architecture combines a wide range of cutting-edge technologies to provide seamless data collection, processing, and analysis while enabling real-time monitoring of gait patterns in AFO wearers. The system employs a surface Electromyography (sEMG) sensor and an Inertial Measurement Unit (IMU) sensor to capture gait pattern data. The sEMG sensor is connected to an Arduino UNO board, while the IMU sensor (MPU-6050) is connected to a NodeMCU ESP-8266 (Espressif Systems, Shanghai, China), as shown in Figure 2.

The data collected by the sEMG and IMU sensors was initially sent to the Arduino, which acts as a data aggregator. The Arduino then transmits the aggregated data to the NodeMCU ESP-8266 via serial communication. Serving as a communication gateway, the NodeMCU ESP-8266 pushes the data to the Cloud layer, where it is stored, processed, and analyzed more efficiently. Once processed, the data are sent for further analysis using machine learning algorithms.

The machine learning model analyzes the data and stores the results in a MySQL database. These results are then made available on the doctor’s devices for further analysis, aggregation, and visualization. Doctors can access this data, review the patient’s gait activity, and provide feedback based on the analyzed results. At the same time, patients can also see those data on their own devices and wait for doctors to confirm and take further measures based on the result.

### 3.3. Participants

Collecting data from patients with ankle–foot issues who wear ankle–foot orthoses (AFOs) was essential for building a reliable machine-learning model. Ensuring the data accurately represents the target population is crucial for refining and improving the device. There were six patients included in the study, all of whom had problems with foot drop. Three of these patients also suffer from cerebral palsy, which exacerbates their disabilities even more. The remaining three patients have foot drop but do not have cerebral palsy, allowing us to compare those with and without additional cerebral palsy diagnoses. The non-progressive motor disorder known as cerebral palsy is characterized by stiffness, involuntary movements, and poor posture [63]. Each patient wore their AFO on their left leg, to which the developed hardware device was attached to collect data. They were instructed to walk for at least four to five minutes, with actual walking times recorded between three to four minutes. The data collected during these sessions were then used to train and develop the machine learning model.

### 3.4. Data Collection and Preprocessing

Implementing machine learning on data collected from the Smart AFO using Inertial Measurement Unit (IMU) and surface Electromyography (sEMG) sensors require careful consideration of several factors. A key aspect is the selection and placement of electrodes for the sEMG sensor to ensure accurate muscle activity data. The gastrocnemius muscle was chosen for this purpose due to its significant role in walking, running, and standing. For accelerometer data, the MPU-6050 sensor was placed on the Lateral Condyle of the Tibia.

Collecting data from patients with ankle–foot issues who wear AFOs was essential for building a reliable machine-learning model. Ensuring the data accurately represents the target population is crucial for refining and improving the device. Six patients, aged between seven and fourteen years (average age nine), participated in the study. Three had a foot-drop condition, while the other three were diagnosed with Cerebral Palsy (as shown in Table 2 for general patient data). Each patient wore their AFO on their left leg, to which the developed hardware device was attached to collect data, and they were instructed to walk for at least four to five minutes, with actual walking times recorded between three to four minutes. The data collected during these sessions were then used to train and develop the machine learning model.

**Figure 3:**
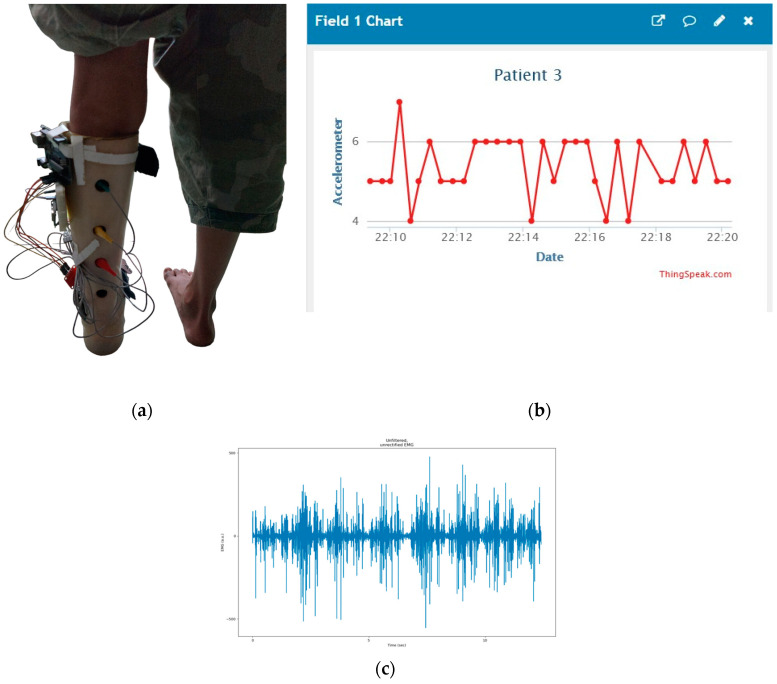
(**a**) Patient’s gastrocnemius muscle data. (**b**) Patient’s accelerometer data plotting. (**c**) Patient’s EMG data plotting.

The sEMG sensor electrodes were placed on the Gastrocnemius muscles to capture electrical activity during movement, as shown in Figure 3a, which is crucial for the study’s focus on foot-drop. Before electrode placement, the skin was thoroughly cleaned to remove dirt, oil, and contaminants, ensuring more accurate and high-quality EMG signals.

With its accelerometer built-in, the MPU-6050 sensor is mounted on the knees, as shown in Figure 1, measured changes in leg direction, providing three-dimensional information along the *X*, *Y*, and *Z* axes. In response to the patient’s walking, an accelerometer was used to record movement data, which were recorded as −6.41 m/s^2^ along the *X*-axis, 4.08 m/s^2^ along the *Y*-axis, and −5.80 m/s^2^ along the *Z*-axis, indicating acceleration in each direction, respectively. A set of raw acceleration values for each session was presented as representative of the entire session as a whole in Figure 3b.

Once the patients began walking with the smart AFO, data collection commenced. All patient data were compiled into a single comma-separated value (CSV) file.

**Figure 4:**
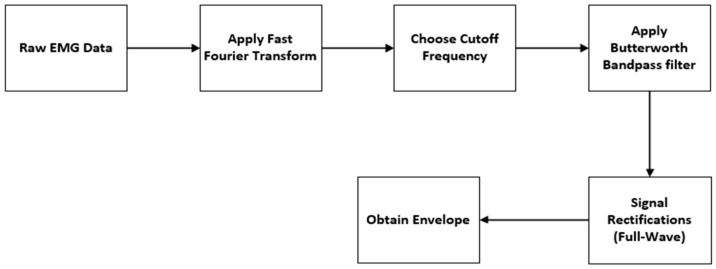
Data Denoising Procedure.

To denoise the unfiltered sEMG data in Figure 3c, the signal processing steps shown in Figure 4 have been followed. The initial step in signal processing involves the conversion of raw data from a time-domain signal to its frequency-domain representation.

Before analyzing the signal in terms of its frequency components, it is essential to preprocess the data to ensure accurate interpretation. Preprocessing involves transforming the signal into the frequency domain using techniques such as the Fast Fourier Transform (FFT). FFT helps identify specific frequency ranges that correspond to different types of noise, such as movement artifacts or other sources of interference. By analyzing the frequency content of the signal, any noise or unwanted components can be identified and removed.

**Figure 5:**
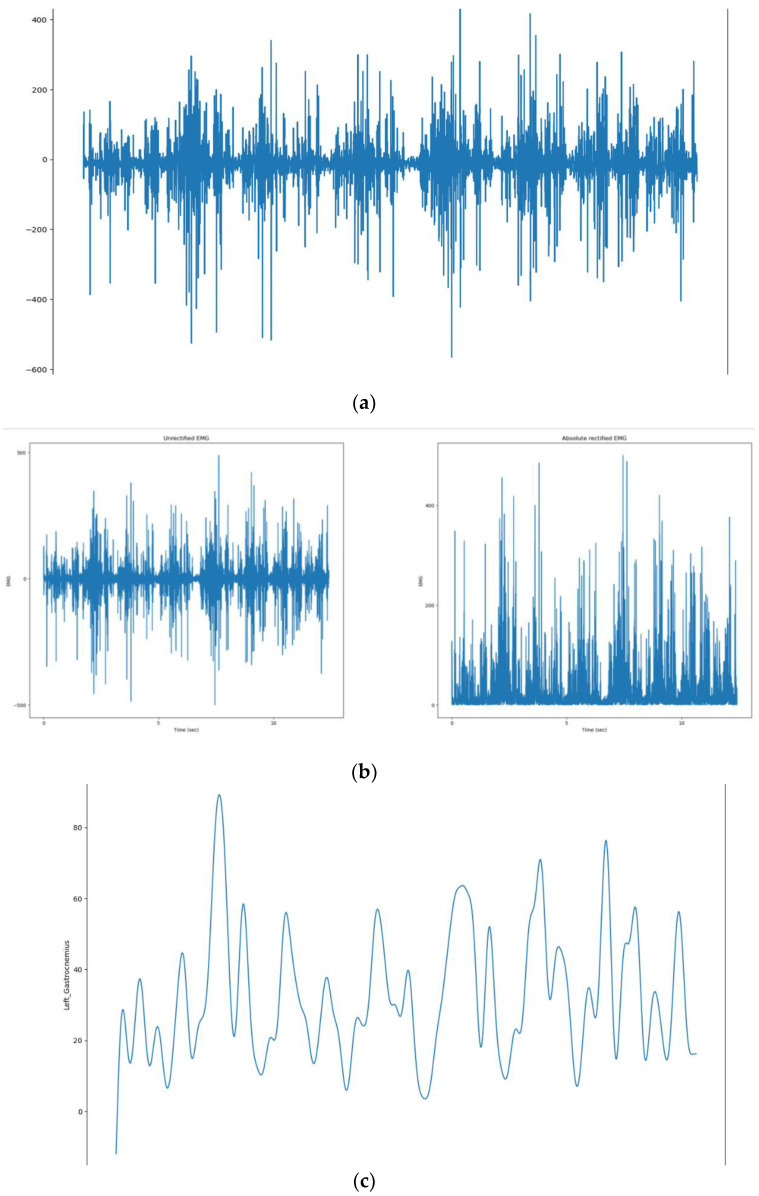
(**a**) Original signal. (**b**) Unrectified and absolute rectified signal. (**c**) Denoised signal.

Based on frequency analysis, a 30 Hz to 450 Hz cutoff frequency range was selected to distinguish between signal and noise components. A low-pass filter with a cutoff at 450 Hz was applied to retain low-frequency signal components while attenuating high-frequency noise. Conversely, a high-pass filter with a cutoff at 30 Hz was applied to keep high-frequency signal components while attenuating low-frequency noise. A fourth-order Butterworth filter was chosen for this purpose because it attenuates signals without introducing significant phase distortion. The fourth-order Butterworth filter strikes an appropriate balance between minimal signal distortion and efficient noise reduction. Following filtering, the signal undergoes full-wave rectification, which takes the absolute value to retain only positive values Figure 5b, effectively eliminating any negative components. Following this, a fourth-order Butterworth low-pass filter with a cutoff frequency of 6 Hz is applied to extract the envelope of the rectified signal. This smoothing process minimizes rapid fluctuations of the rectified EMG signal.

This step is crucial for processing Electromyography (EMG) signals, as it allows for the extraction of the envelope from the rectified signal. Extracting the envelope makes it easier to visualize muscle activity and facilitates further analysis. The envelope helps identify patterns in the signal and quantify the amplitude and duration of muscle contractions, providing valuable insights into muscle performance and functionality Figure 5c.

### 3.5. Training, Validation and Testing

In our study, we utilized a comprehensive clinical dataset comprising 201,527 data rows, carefully split into training, validation, and testing subsets to ensure robust model evaluation and generalization. The dataset was divided into three parts: 70% for training (141,069 samples), 15% for validation (30,229 samples), and 15% for testing (30,229 samples). The training subset allowed the model to learn patterns and relationships within the data through supervised learning. The validation subset was used during training to monitor the model’s performance and fine-tune hyperparameters, helping to prevent overfitting and ensure that the model generalizes well. Finally, the testing subset was reserved for the ultimate evaluation of the model, providing an unbiased assessment of its true capabilities on unseen data. The strategic data-splitting minimized the possibility of data leakage and ensured that the model’s performance metrics accurately reflected its practical relevance.

## 4. Result Analysis

**Figure 6:**
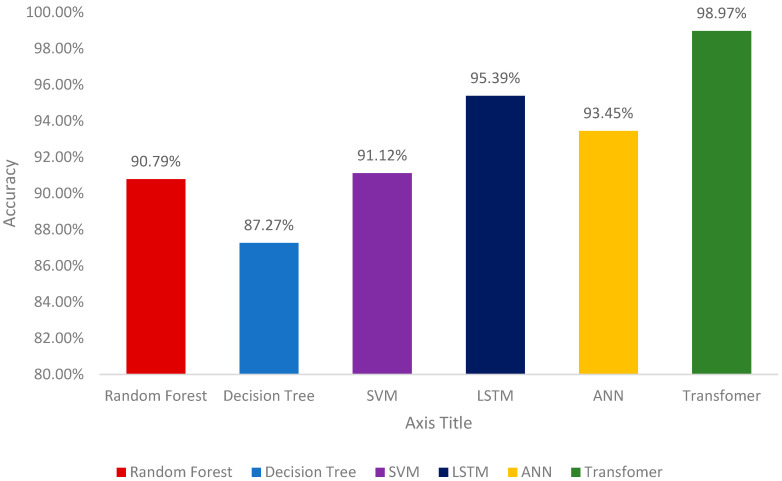
Model Accuracies Comparison.

Figure 6 and Table 3 demonstrate the results of different machine learning models for the identification of gait patterns. The process of identifying gait patterns involves collecting and interpreting data points about an individual’s walking behavior to determine whether it is normal or abnormal. A proper testing and training strategy is essential for validating machine learning models. Therefore, a total of 201,526 samples collected from patients wearing an ankle–foot orthosis (AFO) on their left leg were split into proper training and testing sets. A k-fold cross-validation (k-CV) with k = 3 was adopted for model validation, where samples are randomly selected for training and testing in each fold.

Random Forest model demonstrated solid performance in identifying the gait patterns with an accuracy of 90.79%, precision of 83.78%, and recall of 90.01%. Its F1 score was 89.45%, highlighting a good balance between precision and recall. The Decision Tree model, however, showed a slightly lower performance with an accuracy of 87.27%, precision of 78.59%, and recall of 79.56%. Its F1 score was 79.49%, indicating a relatively lower performance compared to the other models.

The Support Vector Machine (SVM) model achieved an accuracy of 91.12%, with a precision of 90.35% and a recall of 92.98%. The F1 score for the SVM model was 89.67%, which indicates an average performance when compared to the other models tested. The Long Short-Term Memory (LSTM) model performed well, achieving an accuracy of 95.39%, precision of 94.02%, and recall of 93.45%. The LSTM model’s F1 score was 93.46%, showing its strong performance in balancing precision and recall.

The Artificial Neural Network (ANN) model showed excellent results with an accuracy of 93.45%, precision of 97.00%, and recall of 98.32%. Its F1 score was an impressive 97.31%, indicating robust performance [59].

### 4.1. Transformers

A custom Transformer-based model designed for binary classification tasks on clinical datasets. This architecture leverages the power of self-attention mechanisms to capture complex dependencies and relationships within the input data. The input projection layer projects the input features into a higher-dimensional space that matches the model dimension required by the Transformer encoder. Transformers do not inherently understand the order of input sequences. Therefore, positional encodings are added to the input embeddings to provide information about the positions of the tokens within the sequence. These encodings are learned parameters. Multi-Head Self-Attention allows the model to focus on different parts of the sequence simultaneously, learning multiple aspects of the input data. A two-layer fully connected neural network that processes the output from the self-attention mechanism. Layer Normalization and Residual Connections are used after both the self-attention and the Feedforward Network (FFN) to stabilize training and enable better gradient flow. After processing the input through the Transformer encoder layers, global average pooling is applied across the sequence dimension to aggregate the information into a fixed-size vector. This reduces the sequence of embeddings to a single vector that captures the overall context. A fully connected layer reduces the dimensionality of the number of output classes. A sigmoid activation function is used to output probabilities.

In comparison, the Transformer model required some modifications to handle tabular data effectively. Transformers are typically designed for sequential data with fixed input dimensions that must be divisible by the number of attention heads. To adapt the Transformer for tabular data, an input projection layer was added when the input dimension did not meet this requirement. Each data point was treated as a sequence of length one to fit the expected input format for the Transformer.

The Transformer model achieved a high-test accuracy of 98.97%, indicating its superior performance on the dataset. Moreover, it achieved a Precision of 99.02%, a Recall of 98.90%, and an F1 Score of 98.96%, reflecting an impressive combination of identifying positive instances and minimizing false positives. The loss values over the training epochs showed a significant decrease and eventually stabilized, indicating effective training and convergence. The rapid decrease in training loss during the initial epoch underscored the model’s efficient learning capabilities.

The exceptional test accuracy suggests that the Transformer-based model is highly effective in capturing intricate patterns within tabular data. Its performance can be attributed to the multi-head attention mechanism, which allows for robust handling of complex feature interactions. This approach enhances the model’s ability to generalize well to unseen data, ensuring its applicability in various real-world scenarios.

A classification process is not simply a binary process but a sophisticated analysis that gives us actionable insights for patient management. The models are trained on a dataset containing both patient data (labeled as ‘1’) and data from individuals with normal gait patterns (labeled as ‘0’). By dual-labeling, the models learn nuanced differences in gait patterns, which is crucial to understanding the specific needs of patients with gait imbalances. Overall, the results highlight the potential of Transformer-based models in analyzing tabular data. While traditional models like Random Forest, Decision Tree, SVM, Long Short-Term Memory (LSTM), and Artificial Neural Network (ANN) performed admirably, the Transformer-based model outperformed them, demonstrating the advanced capabilities of modern machine learning techniques in dealing with complex datasets. The predictive capabilities of our models stem from this detailed analysis, allowing us to recommend not only whether AFO use is necessary but also how to monitor progress and make timely adjustments to treatment.

### 4.2. Parametric and Non-Parametric Analysis of Results

To evaluate the effectiveness of machine learning models for identifying gait patterns, we conducted both parametric and non-parametric hypothesis tests. Initially, we conducted an Analysis of Variance (ANOVA) test to compare the mean accuracy of the models. The null hypothesis (H0) posited that all models are equally good at identifying gait patterns. We anticipated that if the resulting *p*-value from the ANOVA test was less than 0.05, specifically around 0.01, we would reject H0, indicating that at least one model’s accuracy significantly differs from the others. The alternative hypothesis (H1) suggested that at least one model is better or worse than the others. A significant *p*-value, such as 0.01, would support this claim, confirming the existence of performance differences among the models. We also used the Kruskal–Wallis test, a non-parametric method designed for comparing distributions when normality assumptions cannot be made. The null hypothesis for this test posits that all models perform similarly across different metrics, such as accuracy, precision, recall, and F1 score. If the *p*-value from the Kruskal–Wallis test was less than 0.05, for example, 0.03, we would reject H0, indicating significant differences in the performance distributions among the models. The alternative hypothesis in this context proposes that at least one model demonstrates a different performance pattern. A significant *p*-value, such as 0.03, would support this hypothesis, indicating that the performance metrics vary across the models. Recent research has evaluated the performance metrics accuracy, precision, recall, and F1 score of six machine learning models for the identification of gait patterns: Random Forest, Decision Tree, Support Vector Machine (SVM), Long Short-Term Memory (LSTM), Artificial Neural Network (ANN), and Transformer. The results indicated notable variations in model performance. For instance, the Transformer achieved the highest accuracy at 98.97%, while the Decision Tree recorded the lowest at 87.27%. To determine whether these differences in accuracy were statistically significant, we conducted an Analysis of Variance (ANOVA) test. The ANOVA yielded a *p*-value of 0.005, which is below the conventional threshold of 0.05. This result led us to reject the null hypothesis (H0), indicating that not all models have the same average accuracy. To further investigate which models, differ from one another, we performed post-hoc tests using Tukey’s Honestly Significant Difference (HSD) method. The post-hoc analysis revealed that the Transformer model significantly outperformed the Decision Tree, with a *p*-value of 0.001, and that there was a significant difference between the LSTM and ANN, with a *p*-value of 0.03. Conversely, the comparison between the SVM and Random Forest resulted in a *p*-value of 0.06, suggesting no significant difference in their performances. Additionally, we applied the Kruskal–Wallis test to examine the distribution of performance metrics across the models, which was particularly useful given that our data may not have followed a normal distribution. The Kruskal–Wallis test produced a *p*-value of 0.02, reinforcing our earlier findings by indicating significant differences in performance distributions among the models. To gain deeper insights, we conducted a Mann–Whitney U test to compare the accuracy of Random Forest and Artificial Neural Network models. This test yielded a *p*-value of 0.04, suggesting a significant difference between their performances.

## 5. Limitations

The study encountered challenges in recruiting a sufficient number of participants, influenced by factors such as availability, willingness, and specific inclusion criteria. Consequently, this resulted in a small sample size, which limits the generalizability of the findings. Despite the limited amount of data, the machine learning models did show the best performance and uncovered patterns. Future work will focus on increasing the dataset.

## 6. Conclusions

This paper presents a solution for managing gait imbalances by integrating the Internet of Things (IoT) with machine learning technologies. First, a smart ankle–foot orthosis (AFO) equipped with a surface electromyography sensor (sEMG) and an Inertial Measurement Unit (IMU) was designed for data collection. The collected data were sent to the cloud for storage and further preprocessing. Given the critical importance of input data quality, appropriate signal processing steps were implemented to denoise the data. The denoised data were then provided to several machine learning models, including Random Forest, Decision Tree, Support Vector Machine (SVM), Long Short-Term Memory (LSTM), Artificial Neural Network (ANN), and Transformer models, for the identification of gait patterns. The Transformer model significantly outperformed the other machine learning models for identifying gait patterns, achieving a test accuracy of 98.97% when applied to data collected from six individuals with an average age of nine. The superior performance of the Transformer model can be attributed to its multi-head attention mechanism, which effectively handles complex feature interactions within sensor data.

## Figures and Tables

**Table 1:** Advantages and disadvantages of each technique.

Models	Advantages	Disadvantages
Decision Tree	Decision trees efficiently manage missing data and provide simple, understandable guidelines for making decisions.	Due to their sensitivity to noisy input, decision trees may produce unstable models that vary in response to slight variations in the dataset. They also tend to overfit, especially when dealing with tiny datasets.
Random Forest	They are adaptable for a range of time-series applications since they may be applied to both classification and regression problems.	In time-series data, sequential dependencies are not inherently captured by random forests, so they must be converted into a tabular format to be useful.
Support Vector Machine (SVM)	SVM is well-suited for classification tasks and can be customized using various kernel functions—such as linear, polynomial, and radial basis functions (RBF).	Support Vector Machines (SVM) can be sensitive to parameter choices like kernel type, and they are often memory-intensive.
Long Short-Term Memory networks (LSTM)	When it comes to time-series data, LSTM (Long Short-Term Memory networks) are the most effective.	Computationally intensive, requiring significant resources.
Artificial Neural Networks (ANNs)	ANNs are capable of handling large datasets efficiently and can model non-linear relationships, which makes them versatile for a range of time-series forecasting tasks.	Missing values, outliers, and noise can significantly affect the effectiveness of Artificial Neural Networks (ANNs).
Transformer	Data can be processed in parallel by Transformers, which allows training times to be accelerated significantly.	Transformers require extensive computational resources, especially with regard to memory and processing power.

**Table 2:** Patients’ General Data.

Patient No	Age	Foot-Drop Problem?	Cerebral Palsy?
1	7	yes	yes
2	8	yes	yes
3	9	yes	no
4	9	yes	no
5	10	yes	no
6	14	yes	yes

**Table 3:** Performance Metrics of Models.

Model	Accuracy (%)	Precision (%)	Recall (%)	F1 Score (%)
Decision Tree	87.27	78.59	79.56	79.49
Random Forest	90.79	83.78	90.01	89.45
SVM	91.12	90.35	92.98	89.67
LSTM	95.39	94.02	93.45	93.46
ANN	93.45	97.00	98.32	97.31
Transformer	98.97	99.02	98.90	98.96

## Data Availability

The data presented in this study are available on request from the corresponding author.
